# Connecting the dots: A narrative review of the relationship between heart failure and cognitive impairment

**DOI:** 10.1002/ehf2.15144

**Published:** 2024-10-30

**Authors:** Mauro Massussi, Maria Giulia Bellicini, Marianna Adamo, Andrea Pilotto, Marco Metra, Alessandro Padovani, Riccardo Proietti

**Affiliations:** ^1^ Cardiac Catheterization Laboratory and Cardiology ASST Spedali Civili di Brescia Brescia Italy; ^2^ Department of Medical and Surgical Specialties, Radiological Sciences, and Public Health University of Brescia Brescia Italy; ^3^ Department of Continuity of Care and Frailty, Neurology Unit ASST Spedali Civili Brescia Hospital Brescia Italy; ^4^ Department of Clinical and Experimental Sciences, Neurology Unit University of Brescia Brescia Italy; ^5^ Laboratory of Digital Neurology and Biosensors University of Brescia Brescia Italy; ^6^ Brain Health Center University of Brescia Brescia Italy; ^7^ Liverpool Centre for Cardiovascular Science University of Liverpool and Liverpool Chest and Heart Hospital Liverpool UK

**Keywords:** Brain health, Cognitive impairment, Heart failure

## Abstract

Large clinical data underscore that heart failure is independently associated to an increased risk of negative cognitive outcome and dementia. Emerging evidence suggests that cerebral hypoperfusion, stemming from reduced cardiac output and vascular pathology, may contribute to the largely overlapping vascular dementia and Alzheimer's disease. Despite these insights, cognitive outcomes remain largely overlooked in heart failure management. This narrative review outlines the prevalence and risk of cognitive impairment in heart failure patients, exploring potential shared pathophysiological mechanisms and examining the impact of heart failure therapy on cognitive deficits. Additionally, it discusses clinical implications and suggests future treatment approaches targeting therapeutic outcomes. Cognitive impairment is prevalent among individuals with heart failure, with reported rates varying widely depending on assessment methods. Shared pathological pathways and risk factors, including atrial fibrillation (AF), hypertension, obesity and type 2 diabetes mellitus, suggest a causal link. Mechanisms such as poor perfusion, microembolic events, ischaemic syndromes and cerebral inflammation contribute to this relationship. Moreover, heart failure itself may exacerbate cognitive dysfunction. This emerging understanding posits that vascular dementia and Alzheimer's disease may represent a pathophysiological continuum, driven by both the accumulation of misfolded proteins and cerebrovascular pathology due to cardiovascular dysfunction. Understanding these links is crucial for developing effective treatment strategies. The complex interplay between heart failure and cognitive impairment underscores the necessity for a holistic patient care approach. Both conditions share analogous disease processes, influencing self‐management and independence in patients. Prioritizing brain health in heart failure management is essential to enhance patient prognosis and general well‐being.

## Introduction

Heart failure (HF) is a prevalent condition, affecting at least 26 million people worldwide and posing a significant social and economic impact on public health. Based on a US investigation, healthcare costs for HF vary from $441 to $1585 per‐patient‐per‐month (PPPM), depending on the patient's condition, whether stable or experiencing a worsening HF event.^1^ This burden is expected to increase as the global population ages, with HF prevalence predicted to double within 40 years.^2^ Similarly, mild cognitive impairment (CI), defined as a statistical construct denoting performance on cognitive tests consistently below age‐ and education‐specific norms,^3^ and its severe form, dementia, are also associated with increasing social and economic burdens. Current estimates suggest that over 55 million people worldwide are affected by CI, though many mild cases may go unrecognized.^4^ In a German Health Economics study, the net annual costs of dementia vary by disease stage, ranging from €15 474 up to €41 808.^5^ Similar to HF, the prevalence of CI is higher in Western countries, primarily due to the older average age of the population. However, it is important to recognize that the lower prevalence of CI in middle‐ and low‐income countries may also be attributed to underdiagnosis, in addition to differences in population age. With the population aging in these regions as well, the number of individuals affected by dementia is projected to nearly double every 20 years worldwide, with a significant increase expected in developing countries.^6^ CI and HF often co‐occur in the aging population and they probably interact each other, even if the precise pathophysiological processes underlying this association are not yet fully understood. There is recognition that HF may be considered an independent predictor, though the universality and unequivocal nature of this prediction remain subjects of ongoing research.^7^

This is a non‐casual association: a significant portion of dementia cases can be attributed to cardiovascular risk factors, which could potentially be prevented through cardiovascular risk modification. Furthermore, HF can lead to varying degrees of CI, influenced by genetic factors (e.g., apolipoprotein E (APOE) and environmental elements. Hence, given the presence of several of the nine ‘aspects of non‐casual association’ discussed by Hill in 1965 (strength of association, consistency, specificity, temporality, biological gradient, plausibility, coherence, experiment and analogy), we can hypothesize a multifaceted relationship between HF and CI.^8^

In this narrative review, we will firstly describe the prevalence and analyse the risk of CI, in patients with HF. Second, we will discuss the possible pathophysiological mechanisms by which HF constitutes a risk factor for CI. Third, we will summarize the literature evidence on the effects of HF therapy on cognitive dysfunction. Finally, we discuss clinical implications and future treatment approaches in terms of therapeutic targets.

## Prevalence of cognitive impairment in heart failure

According to the existing literature, CI is highly prevalent among individuals with HF, with reported rates spanning from 3% to 80%, depending on the assessment methods for cognition (*Table* *1*). Studies drawing from extensive cohorts, including data from International Classification of Diseases (ICD) records, consistently report a prevalence of almost 15% with some single study reporting different values. For instance, Lafo et al. observed a 36% prevalence of CI among a highly co‐morbid population of veterans hospitalized primarily for HF; this population had also a notably high rate of hospital readmission (30‐day readmission rate: 15%, 1‐year readmission rate: 59%) and mortality (30‐day mortality 6%, 1‐year mortality 41%).^9^ Biagi et al. reported a 35% prevalence in another extensively co‐morbid cohort of 1444 patients admitted with a diagnosis of chronic HF to Internal Medicine Department.^10^ Conversely, three studies reported a lower dementia prevalence (3.2%, 6.9% and 6.9%, respectively) considering a relatively low co‐morbid population.^11^, ^12^, ^13^ Unfortunately, for the majority of these cohort studies the clinical subtypes of dementia/CI (vascular dementia vs. Alzheimer's disease (AD) are not available. The only published prospective data on mild CI (MCI) and HF originates with fully data available have been presented within the COGNITION.MATTERS HF study, following 148 patients for up to 6 years, collecting data on cognitive function, brain imaging and inflammatory biomarkers.^14^ The study's principal findings revealed that HF patients exhibited selective deficits in attention (41%) and verbal memory domains (28%), with medial temporal lobe atrophy (potentially indicating underlying AD‐related pathology) identified as a probable structural correlate of cognitive dysfunction. Studies investigating the potential risk of CI development in HF patients remain limited and yield conflicting results (*Table* *2*). Most studies suggest a significantly higher risk compared to the general population (with HR ranging from 1.3 to 1.7), while a few show non‐significant outcomes, possibly due to the relatively low number of HF patients in those studies.^15^, ^16^, ^17^ In contrast, a Swedish nationwide analysis reported a potentially protective role of HF against dementia risk in patients with atrial fibrillation (AF),^18^ hypothesizing that effective drug treatment in HF patients and higher mortality rates in the AF and HF subgroup might explain this unexpected result. A comprehensive meta‐analysis by J.A. Cannon et al. involving 37 studies, demonstrated a significantly elevated risk of CI in HF patients (prevalence 43%) compared to matched controls without HF.^19^ It is worth noting that patients affected by CI are often excluded from clinical studies, which may underestimate the actual prevalence. Included studies, however, exhibit significant heterogeneity, with dementia prevalence ranging from 10% to 79%. Another meta‐analysis by N. Li, which encompassed 119 studies, reported a pooled proportion of 41% in the 95 studies that examined CI using standardized tests.^20^ Several studies have explored the timing of cognitive decline in relation to the onset of HF. Most of these studies suggest that cognitive decline tends to manifest after the onset of HF rather than before it. For example, Sterling et al. found that the prevalence of CI 1–18 months before HF onset was comparable to that in control subjects (14.9% [11.7–18.6%] vs. 13.4% [11.6%–15.4%], *P* < 0.43).^21^ Similarly, Sun et al. reported a low prevalence of dementia in patients with newly diagnosed HF (4.2% in women and 2.5% in men).^11^ Regarding the impact of HF duration on cognitive decline, Hammond et al. reported a faster decline in Modified Mini‐Mental State Examination (MMSE) scores over 5 years following HF onset.^22^ Bressler et al. also found that the greatest six‐year decline in cognitive test scores was significantly associated with an increased risk of developing HF.^23^ In the ARIC study, Witt et al. observed that participants with HF had a higher prevalence of dementia (RRR = 1.60 [95% CI 1.13, 2.25]) and MCI (RRR = 1.36 [95% CI 1.12, 1.64]) at visit 5 (2011–2013), with a decline in cognition between visit 4 (1996–1998) and 5 that was greater in those who developed HF after visit 4.^24^ Long‐term data from Adelborg et al. indicated an increased risk of dementia in patients with HF over 1–35 years of follow‐up, with risk ratios of 1.21 (95% CI 1.18–1.24) during the first 10 years, 1.19 (95% CI 1.11–1.28) during 11–20 years, and 1.38 (95% CI 1.07–1.79) during 21–35 years.^25^ Ren et al. further reported that 11.0% of patients developed new‐onset dementia after a median follow‐up of 4.1 years (IQR: 1.2–10.2) post‐HF diagnosis, with a higher incidence in women (64%).^26^ Regarding the correlation between the severity of HF and the prevalence of CI, several studies have demonstrated a significant relationship. Lee et al. found that patients with New York Heart Association (NYHA) functional class II or higher were independently associated with cognitive decline.^27^ Similarly, Brunén et al. confirmed that patients with NYHA class III–IV had a higher prevalence of CI.^28^ The WARCEF trial highlighted an independent association between left ventricular ejection fraction (LVEF) and MMSE decline.^29^ On the other hand, the ARIC cognition study found no significant difference in the incidence of CI between HF with reduced ejection fraction (HFrEF) and HF with preserved ejection fraction (HFpEF) patients.^24^ Additionally, in acute decompensated HF, no significant disparity was observed in mean Montreal Cognitive Assessment (MoCA) scores or in the proportion of patients with MoCA scores below 26 between HFpEF and HFrEF groups.^30^ The link between LVEF and cognitive function may be nonlinear, suggesting a potentially exponential association, with a stronger impact observed at lower LVEF levels compared to higher ones. Lastly, the potential misdiagnosis of depressive symptoms as cognitive impairment in older HF patients must be considered, as this could affect the accuracy of CI prevalence estimates. Some studies, have noted the difficulty in differentiating between CI and depression in these patients, highlighting the need for careful clinical evaluation.^27^

**Table 1:** Epidemiological evidence on prevalence of dementia in patients with heart failure

Study	Sample size of HF patients (*N*)	Mean age	Prevalence of cognitive impairment	Cognitive impairment definition
Ahluwalia, 2012 (USA)	18 332	81 (75–86)	21.3%	CCW
Brunén, 2021 (ES)	3845	79 ± 9	16%	SPMSQ
Biagi, 2014 (IT)	1444	79 ± 9	35%	Pfeiffer test
Chitnis, 2015 (USA)	8062	74 ± 9	14.1%	ICD‐9
Cancian, 2013 (IT)	1905	78 (65–80)	15.7%	GP data sheet
Carter, 2016 (UK)	31 760	74 ± 14	6.8%	ICD‐10
Fujiki, 2022 (JPN)	1852	76 ± 8	6.9%	Previous medical reports
Yaku, 2018 (JPN)	3555	80 (70–90)	16.8%	Physician records
Lafo, 2022 (USA)	21 655	77 ± 10	36%	ICD‐9
Lee, 2019 (International)	1846	61 ± 11	13.6%	≥2 MMSE points drop
Frey, 2018 (GER)	148	65 ± 10	41%	Test Battery of Attentional Performance
Sterling, 2019 (USA)	436	70 ± 9	14.9%	SIS
Sun, 2017 (CAN)	90 707	73 ± 13	3.2%	ICD‐10

Abbreviations: CCW, Chronic Condition Warehouse; GP, general practitioner; ICD, International Classification of Disease; MMSE, Mini‐Mental State‐Examination; SIS, Six‐Item Screener; SPMSQ, Short Portable Mental Status Questionnaire.

**Table 2:** Epidemiological evidence on incidence of dementia in patients with heart failure

Study	Sample size (*N*)	HF prevalence	Mean age	Cumulative incidence (%)/hazard ratio of cognitive impairment in HF patients (follow‐up duration)	Cognitive impairment definition
Aldeborg, 2017 (DK)	1 946 497	16.7%	77 (69–84)	1.21 (95% CI 1.18–1.24) (6 years)	ICD
Bressler, 2017 (USA)	9895	12.4%	59 ± 6	1.43 (95% CI 1.24–1.66)	DSST
				1.43 (95% CI 1.24–1.66)	DWRT
				1.12 (95% CI 0.98–1.28) (6 years)	WFT
Cacciatore, 1998 (IT)	1075	8%	75 ± 7	1.96 (95% CI 1.07–3.58)	MMSE
de Bruijn, 2015 (NL)	7003	3%	69 ± 9	0.87 (95% CI 0.59–1.28) (8 years)	DSM‐III
Hammond, 2018 (USA)	4864	10.2%	76 ± 6	1.70 (95% CI 1.2–2.2) (5 years)	MMSE; DSST
Haring, 2013 (US)	6455	1%	73 (60–84)	1.49 (95% CI 0.73–3.04) (8.4 years)	MMSE, neurocognitive and neuropsychiatric objective exam
Legdeur, 2019 (NL)	442 428	6.8%	87 (65–115)	1.26 (95% CI 1.06–1.49) (3.6 years)	GP records
Noale, 2013 (IT)	2501	5%	71 ± 5	1.40 (95% CI 0.5724–3.4532) (7.8 years)	MMSE; ICD‐10; DSM III‐R
					NINCDS‐ADRDA; CAMDEX; Pfeiffer test; neurological examination
Qiu, 2006 (SW)	1301	15%	83 ± 5	1.70 (95% CI1.24–2.34) (5 years)	MMSE
Peters, 2009 (UK)	3336	0.4%	>80	0.55 (95% CI 0.08–3.91) (1.8 years)	MMSE <24 or drop ≥3 point
					DSM‐IV; CERAD
Rusanen, 2014 (FI)	1510	6%	50 ± 6	1.40 (95% CI 1.01–1.93) (25 years)	DSM‐IV; NINCDS‐ADRDA
				0.87 (95% CI 0.35–2.16) (8 years)	
Wändell, 2018 (SW)	537 513	39%	77 ± 9	0.79 (95% CI 0.76–0.84) (4 years)	ICD‐10
Witt, 2018 (USA)	6495	14.7%	76 ± 5	1.60 (95% CI 1.13–2.25) (15 years)	CDR; WMS‐III, WAIS‐R; DSB, BNT, MMSE

Abbreviations: BNT, Boston Naming Test; CAMDEX, Cambridge Mental Disorders of the Elderly Examination; CDR, Clinical Dementia Rating; CERAD, Consortium to Establish a Registry for Alzheimer's Disease; DSM, Diagnostic and Statistical Manual of Mental Disorders; DSST, Digit Symbol Substitution Test; DWRT, Delayed Word Recall Test; ICD, International Classification of Disease; MMSE, Mini‐Mental State Examination; NINCDS‐ADRDA, National Institute of Neurological Communicative Disorders and Stroke – Alzheimer's Disease and Related Disorders Association; WAIS‐R DSB, Digits Span Backward of Wechsler Adult Intelligence Scale‐Revised; WFT, Word Fluency Test; WMS‐III, Wechsler Memory Scale‐III.

## Cardiovascular risk factors and cognitive impairment

**Figure 1:**
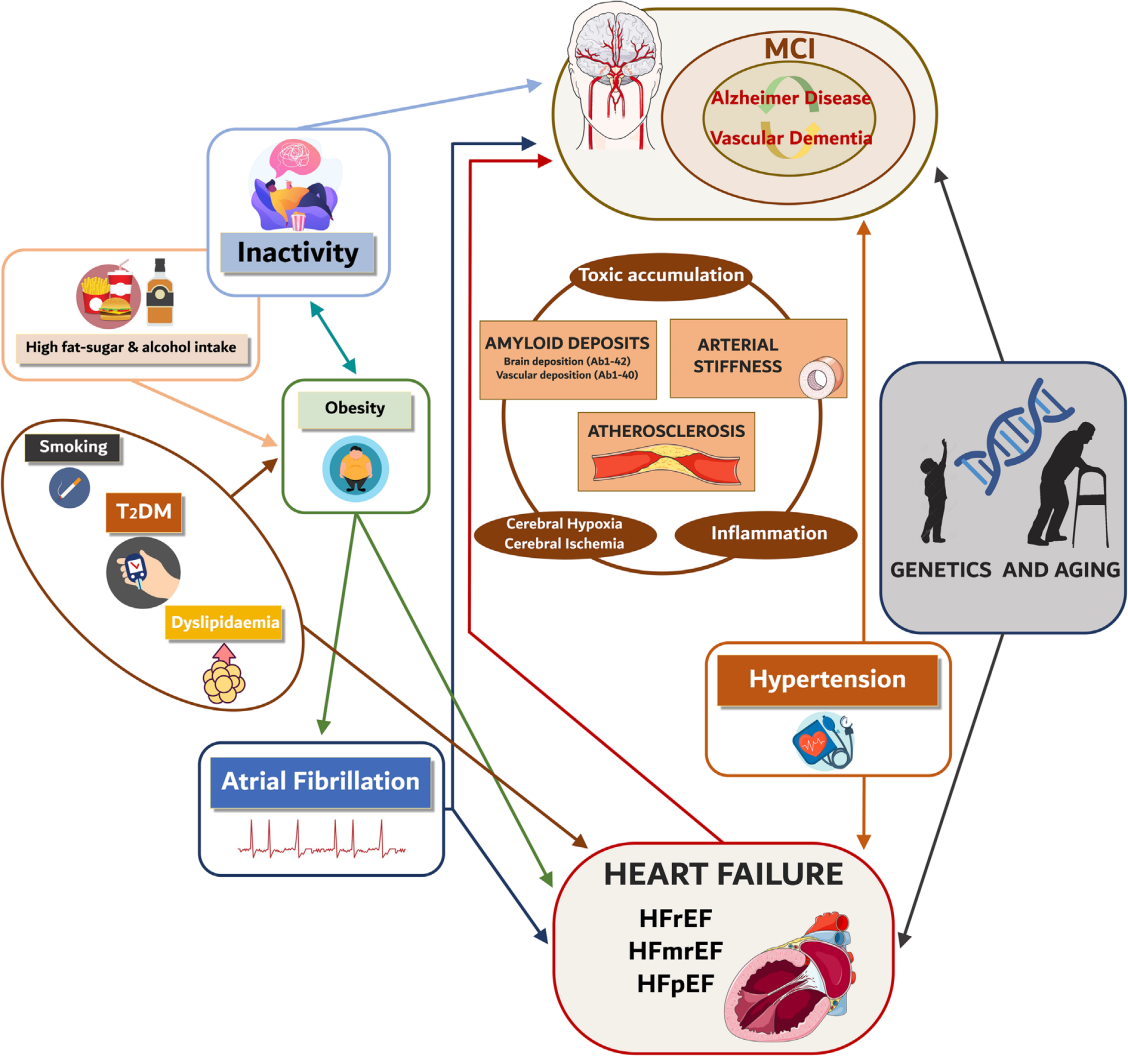
Schematic diagram mapping the shared risk factors and underlying physiological mechanisms between heart failure and cognitive dysfunction.

Evaluation of the complex interaction between the heart and brain is necessary to address patient prognosis and well‐being. The shared pathological pathways and risk factors, including AF, hypertension, obesity and type 2 diabetes mellitus (T2DM), provide vital clues to understanding the causal link between HF and CI.^31^, ^32^ Poor perfusion, microembolic events, ischaemic syndromes, cerebral inflammation, endothelial dysfunction with blood–brain barrier damage, and the presence of amyloid deposits may collectively contribute to unravelling the intricate relationship between HF and CI (*Figure* *1*).

### Atrial fibrillation and cognitive impairment burden

The age‐adjusted incidence rate of AF, reported as 1.33 per 1000 person‐years, exhibits a range from 0.13 per 1000 person‐years in individuals aged under 55 years, to 7.65 per 1000 person‐years in those aged 85 years or older.^33^ Multiple observational studies, along with several comprehensive meta‐analyses, have consistently highlighted that AF is closely linked to an elevated risk of CI and dementia. Notably, the extensive biracial population‐based ARIC‐NCS study, spanning two decades, found that individuals diagnosed with AF exhibited a substantially greater cognitive decline when compared to their counterparts who did not develop AF (HR 1.23, 95% confidence interval (CI) 1.04–1.45).^34^ Notably, even after rigorous adjustment for common confounding factors such as age, sex, education, apolipoprotein E, smoking, body mass index (BMI), arterial hypertension, T2DM, coronary heart disease and stroke, the association remains significant, especially among younger patients. This underscores the autonomous role of AF in accelerating cognitive dysfunction, prompting further inquiry into its potential causal contribution. The primary driver of AF‐induced CI is cerebral infarction. However, other proposed pathways include:

- *Cerebral hypoperfusion*: AF unsettles the heart's atrioventricular synchrony, leading to decreased cardiac output, stroke volume and blood pressure. Research in elderly populations with persistent AF has revealed a connection to reduced total cerebral blood flow and impaired whole brain perfusion, as assessed by phase contrast Magnetic Resonance Imaging (MRI) of the brain.^35^
- *Inflammation and systemic atherosclerotic vascular disease*: inflammation is believed to promote hypercoagulability and thrombus formation, potentially increasing the risk of stroke and disrupting cerebrovascular regulation, which has links to AD and vascular dementia. Inflammation may also serve as a nonspecific marker of atherosclerotic vascular disease. Recent studies have further explored the connection between inflammation, atherosclerosis and AF, highlighting the potential impact on cognitive decline.^36^
- *Microhemorrhage*: the relationship between the burden of cerebral microbleeds, often attributed to oral anticoagulant therapy, particularly in lobar locations (overlapping with cerebral amyloid angiopathy, CAA), and cognitive function is a subject of ongoing debate.^37^, ^38^

Recent evidence suggests that rhythm‐control strategies, particularly catheter ablation, are associated with a reduced risk of CI and dementia in patients with AF. A meta‐analysis by Guo et al. found that rhythm‐control therapy was significantly associated with a lower risk of future dementia (HR 0.74, 95% CI 0.62–0.89) compared to rate‐control strategies. Specifically, AF ablation was linked to significantly lower risks of overall dementia (HR 0.62, 95% CI 0.56–0.68), AD (HR 0.78, 95% CI 0.66–0.92) and vascular dementia (HR 0.58, 95% CI 0.42–0.80).^39^ Notably, an American study of 38 176 patients demonstrated that catheter ablation was associated with a 41% lower risk of dementia compared to antiarrhythmic drugs (1.9% vs. 3.3%; HR 0.59, 95% CI 0.52–0.67, *P* < 0.0001).^40^ Similarly, a nationwide cohort study in Korea involving 11 726 AF patients reported a reduced risk of dementia with catheter ablation compared to antiarrhythmic or rate‐control drugs alone (HR 0.73, 95% CI 0.58–0.93).^41^

### Hypertension and cognitive impairment burden

Hypertension affects approximately 1.4 billion adults, 31% of the adult population, worldwide.^42^ Recent findings from a comprehensive meta‐analysis conducted by Qin and colleagues revealed a noteworthy association between hypertension and MCI, with an overall pooled prevalence of 30%. Interestingly, MCI prevalence in hypertensive patients demonstrated regional disparities, with rates of 26% in Asia, 40% in Europe and 17% on a global scale. After age‐stratification, the prevalence of MCI was 44% (95% CI 1–86) in hypertensive patients under 60 years old compared to 28% (95% CI 24–32) in those aged 60 and above. It is important to note that the younger age group's data is based on only two studies, leading to a wide confidence interval.^43^ Mehra et al. reported a notably high prevalence of 66% for MCI in their study on the impact of hypertension on cognitive functions. In this study, 45.7% of participants had metabolic syndrome, a rate significantly higher than in the general population (9.2%–41%). Those with metabolic syndrome exhibited poorer cognitive performance across all domains of the MoCA, even after adjusting for age, education, depression severity and illness duration. Lower education levels, lower income and higher age were significantly associated with lower MoCA scores.^44^ Additionally, the use of MoCA, which may lack precision in individuals with lower education levels, could contribute to the high prevalence reported.^45^ Hypertension might play a pivotal role in the pathophysiology of CI, with a multifaceted impact on vascular structure. The potential connection between hypertension, HF and CI^46^ is driven by a complex interplay of mechanical, cellular and molecular factors that trigger vascular smooth muscle cell remodelling.^47^ Hypertension fosters the development and accumulation of atherosclerotic plaques in key arteries, including the carotid, vertebral and intracranial cerebral arteries. It is closely associated with or often preceded by arterial stiffening, attributed to various factors such as collagen deposition and elastin fragmentation. This stiffening elevates pulse pressure and enhances mechanical stress transmission through the cerebrovascular system, leading first to small vessel adaptive changes aimed at protecting the downstream microcirculation, and then to small penetrating arteries fibrotic thickening that are a common feature in both HF and dementia. Furthermore, microvascular rarefaction, which involves a reduction in vascular density encompassing both capillaries and arterioles, is observed in both human subjects and animal models of hypertension.^48^, ^49^ It is believed to result from the increased pressure transmitted to the microvascular bed. Given the limited presence of vessels in the white matter, this phenomenon may contribute to the development of white matter lesions. Cerebral microhaemorrhages are closely associated with hypertension^50^, ^51^ and are linked to compromised cognitive function. The progression of white matter hyperintensities and small vessel disease appears to correlate in several longitudinal studies with the duration of hypertension and the poor effectiveness of blood pressure control.^52^ Another characteristic of small vessel disease involves the enlargement of the perivascular space surrounding intracerebral arteries and veins.

### Obesity, type 2 diabetes mellitus and cognitive impairment burden

In a nationwide survey conducted by the National Health and Nutrition Examination, the age‐adjusted prevalence of obesity in the US was recorded at 42.4% in 2017–2018, demonstrating variations across age groups: 40.0% for individuals aged 20–39, 44.8% for those aged 40–59, and 42.8% for adults aged 60 and above.^53^ Additionally, a robust, dose‐dependent relationship has been established, connecting higher BMI levels with an increased risk of HF, particularly in patients with HFpEF.^54^, ^55^ A multitude of deleterious pathological features, such as insulin resistance, gut dysbiosis, oxidative stress, inflammasome activation and systemic inflammation, are associated with obesity and T2DM.^56^, ^57^, ^58^ Each of these pathological features may contribute to neuroinflammation and brain injury. Chronic systemic inflammation is a hallmark of obesity and can be instigated by adipose tissue expansion (adipocyte hypertrophy and proliferation). Adipose tissue expansion promotes a hypoxic environment where adipocytes undergo apoptosis, causing further inflammation.^59^ Adipokines are of particular interest due to their ability to modulate insulin resistance, dysregulate the gut‐brain axis, and increase systemic inflammation, which may contribute to the development of neuroinflammation and dementia pathology.^60^ Impaired insulin signalling may be one of the early drivers of amyloid deposition in AD, showing how this morbidity can link the pathology of T2DM and AD. Research has shown that insulin degrading enzyme can break down amyloid beta (Aβ), indicating that insulin resistance could potentially contribute to changes in Aβ metabolism and increased amyloid pathology in AD.^61^ Insulin resistance in murine models has been shown to promote amyloid precursor protein (APP) phosphorylation and to increase the formation of amyloid plaques in the brain^62^; moreover, insulin resistance has been shown to contribute to neuroinflammation and neurodegeneration.^63^, ^64^

### Physical inactivity and cognitive impairment burden

It is crucial to underscore the pivotal role of physical inactivity in patients with HF, particularly in the context of cognitive function. Studies have compellingly demonstrated that physical inactivity is significantly associated with CI in HF patients, in terms of executive function, attention, processing speed and cognition screening scores.^65^ This evidence emphasizes the critical need for interventions aimed at reducing sedentary behaviour and increasing physical activity levels. Engaging in regular physical activity not only improves cognitive function but also holds potential for reducing depression and enhancing overall well‐being.^66^ Higher levels of physical activity, measured in terms of step count and time spent in moderate‐vigorous activity, have consistently shown positive correlations with improved cognitive function, while lower physical activity levels have been associated with cognitive dysfunction.^67^

### Hyperlipidaemia and cognitive impairment burden

Atherosclerosis, the hallmark of hyperlipidaemia, is a systemic process that affects large and small blood vessels throughout the body, including the brain. Furthermore, the brain is highly dependent on cholesterol for membrane structure and function. Dysregulation of cholesterol metabolism has been linked to CI, and it has been suggested that cholesterol‐lowering therapies could reduce the risk of cognitive dysfunction.^68^, ^69^

## Translational implications

The connection between HF and CI extends beyond shared risk factors; HF itself can potentially contribute to cognitive dysfunction. Complex interactions occur at multiple levels between AD hallmarks, such as extracellular senile plaques rich in Aβ peptide, intraneuronal neurofibrillary tangles (NFTs) composed of hyperphosphorylated microtubule‐binding protein tau (p‐tau), and key CV disease features, including neuroinflammation, cerebrovascular dysfunction, blood–brain barrier (BBB) injury and cerebral amyloid angiopathy (CAA).^3^, ^70^ Recent evidences suggests that AD and cerebrovascular dementia are part of a disease continuum, where intersecting pathways can favour either vascular or parenchymal amyloid deposition. The primary amyloid peptide in parenchymal lesions of AD is Aβ1–42, while Aβ1–40 is more prevalent in peripheral atherosclerotic lesions. Factors altering the Aβ1–40/−42 ratio, like APOE, favour amyloid deposits in the form of cerebral amyloid angiopathy rather than parenchymal plaques. This preference for Aβ species in different tissues may result from substantial Aβ1–40 production by platelets, plaque‐invading macrophages, endothelial cells and vascular smooth muscle cells. Additionally, *APOE* isoforms have varying effects on Aβ production, aggregation and clearance.^71^ Specifically, the APOEe4 allelic variant represents the most potent genetic risk factor for sporadic AD and identifies a distinct clinicopathological entity, while APOEe2 is associated with a lower AD risk.^71^ Nevertheless, evidence suggests that APOEe4 is linked to compromised cerebrovascular integrity and function, contributing to blood–brain barrier dysfunction and serving as a risk factor for CI due to cerebrovascular dementia, both in the presence and absence of AD pathology.

### Heart failure pharmacotherapy and cognitive impairment

The effect of pharmacological therapy for HF on cognitive dysfunction has been a topic of investigation, with specific focus on certain medications. While anticoagulation's established role in reducing incident dementia in AF patients is well‐known, its impact on HF patients without known AF remains uncertain. Experimental studies suggest that anticoagulant agents, such as heparin and enoxaparin, may inhibit amyloid beta neurotoxic effects through their glycosaminoglycan structure, affecting APP function and BACE1 activity.^72^ The evidence regarding their effect on cognitive test performance and covert infarcts in stable CAD or peripheral artery disease patients treated with rivaroxaban and aspirin is inconclusive.

ARNIs are theorized to potentially impact cognitive function by affecting Aβ peptides in the central nervous system. Neprilysin inhibition could reduce their breakdown, while increased bradykinin levels may damage the BBB and contribute to amyloid plaque deposition.^73^ The real‐world analysis of the PARADIGM‐HF trial did not demonstrate these effects.^74^ Similarly, inhibition of angiotensin‐converting enzyme (ACE) may influence the availability of amyloid beta peptides. ACE inhibitors (ACEIs) have been found to increase levels of Ab1–42, while the results for Ab1–40 levels have shown inconsistency, with either an increase or no change observed. While pathophysiological hypotheses and in‐vitro studies suggest potential mechanisms, clinical data also managed to shed light on this matter. For instance, in a study involving 1220 patients admitted with HF, abbreviated mental test scores improved from admission to discharge in 30% of patients after the initiation of ACEIs, compared to 22% of HF patients not receiving these drugs (odds ratio, 1.6 [95% CI, 1.2–2.1]).^75^ The role of sodium‐glucose cotransporter‐2 inhibitors (SGLT2Is) is also being explored; in a prospective study of 162 frail patients with diabetes, HFpEF and baseline MoCA scores <26, empagliflozin monotherapy correlated with improved MoCA scores 1 month after admission, whereas treatment with insulin or metformin did not. It is important to note that the authors did not control for treatment duration or serum glucose levels.^76^ These findings suggest a potential cognitive benefit associated with SGLT2Is in HF patients, and further research is needed to elucidate the mechanisms behind these effects and to explore the long‐term cognitive implications of this therapy. Recent studies have shown promising outcomes for glucagon‐like peptide‐1 receptor agonists (GLP‐1 RAs) in obese patients with HFpEF.^77^ Four meta‐analyses have been conducted to assess the influence of GLP‐1 RAs on cognitive function in individuals with T2DM.^78^, ^79^, ^80^, ^81^ However, it is important to note that none of these analyses specifically investigated the cognitive effects in patients with HF, highlighting the need for further research to better understand the potential effects of GLP‐1 RAs on cognitive function.^82^ Despite the significance of CI potentially linked with HF, major pharmacological trials, including those involving ARNIs and SGLT2Is, have not extensively reported on this aspect. It may not be feasible to include dementia patients in these trials, but the inclusion of patients with MCI could provide valuable insights, considering the potential impact on adherence and treatment efficacy.

## Clinical implications of MCI in patients affected by heart failure

CI and brain health in HF patients have profound clinical implications, evident in a meta‐analysis of over 10 000 individuals, highlighting reduced treatment adherence, compromised self‐care abilities and a decreased likelihood of seeking assistance among those with CI.^83^ While HF guidelines underscore the importance of recognizing cognitive impairment, especially in the context of frailty, communication challenges and end‐of‐life decisions, they currently lack specific recommendations for routine screening or diagnosis in clinical practice. Additionally, insights from the Registro de Insuficiencia Cardiaca (RICA) suggest that HF patients with severe CI face heightened mortality and morbidity risks, characterized by advanced age, increased co‐morbidity burden, lower survival rates and a higher incidence of death or readmission at 1 year.^28^ Nevertheless, acknowledging this association is crucial as it influences the risk/benefit ratio of various therapeutic interventions. The efficacy of pharmacotherapy and procedural interventions may be constrained if patients have limited life expectancy to derive benefits. For instance, if life expectancy is less than 1 year, the probability of benefit from interventions such as implantable cardioverter‐defibrillators or transcatheter mitral/aortic valve procedures is low, and hence not warranted.^84^

Beyond clinical implications, the economic impact of cognitive impairment in HF warrants attention, emphasizing the need for a comprehensive bioeconomical assessment to address the multifaceted challenges posed by this condition.

To address a promptly diagnose of MCI in patients with HF, van Nieuwkerk and colleagues have proposed a two‐step algorithm.^85^ The first step involves inquiring about substantial cognitive decline noted by patients or their relatives over the past year, followed by assessing its impact on daily life. A positive response triggers cognitive screening, as some patients with severe deficits may not perceive their cognitive problems. Clinical suspicion, raised by unexplained falls, medication errors, history of delirium, or depressive symptoms, also warrants screening, especially in patients over 65. The second step involves a multidomain cognitive screening test tailored to the patient's baseline cognitive level. Given that executive functioning and memory are commonly affected in patients with cardiac conditions, screening tools should cover these domains. The MoCA and MMSE are commonly used for global cognition assessment. MMSE, with a cut‐off of 24 points, exhibits good sensitivity and negative predictive value but lower specificity for dementia. MoCA, designed for MCI, is more sensitive but has lower specificity for dementia.^86^ An optimal MoCA cut‐off may be 26, showing excellent sensitivity and negative predictive value but poorer specificity for dementia. The Informant Questionnaire on Cognitive Decline in the Elderly (IQCODE) is useful for proxies in assessing cognitive trajectories.^74^ Multidomain cognitive screening tool, such as MoCA or the MMSE or similar digital cognitive testing^87^ stratify risk and inform the need for referral to specialist memory services. HF patients might be affected by multiple domains CI (including attention, executive function, language, memory and visual–spatial capacities). The inclusion of standardized neurocognitive outcomes within cardiovascular trials could serve as a beacon for identifying high‐risk patients. NeuroARC, with its commitment to standardized neuropsychological endpoint definitions, advocates for the integration of cognitive screening at each trial visit, possibly leveraging the MoCA as a valuable tool.^88^ The current landscape lacks reliable imaging and blood biomarkers for identifying individuals at risk for AD or cognitive decline.^89^, ^90^ Integrating such biomarkers into screening strategies could significantly advance the identification of high‐risk patients and improve overall cognitive assessment in the HF population.^76^

## Management

**Figure 2:**
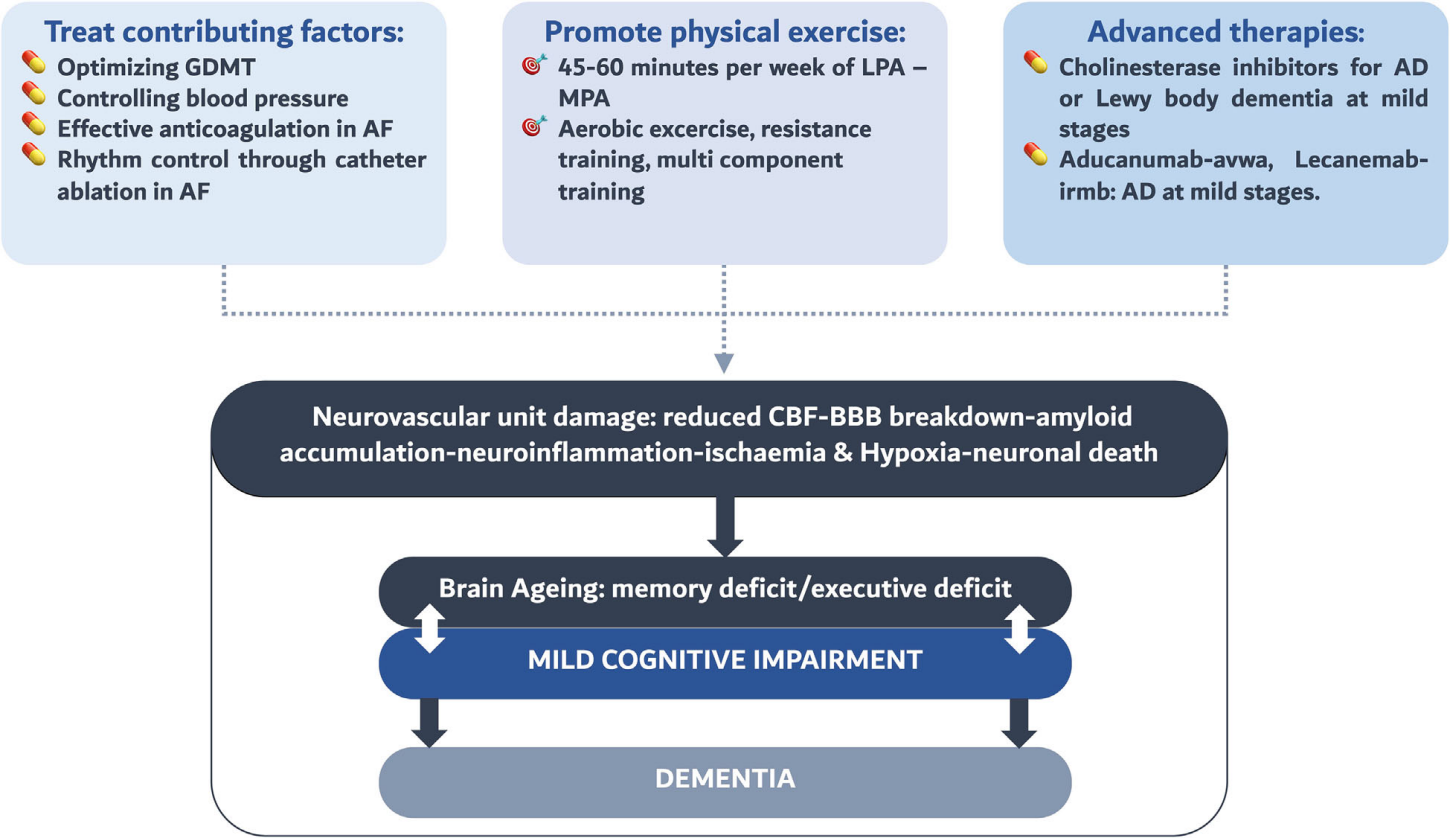
Potential approaches for managing cognitive dysfunction in heart failure patients. This diagram outlines various pharmacological and non‐pharmacological strategies for addressing cognitive dysfunction in individuals with heart failure. GDMT, guideline‐directed medical therapy; LPA, low‐intensity physical activity; MPA, moderate‐intensity physical activity.

A recent statement from the American Heart Failure Society highlights that there are no specific interventions proven to improve cognition or delay the progression of CI in patients with HF.^91^ Potential approaches include the treatment of contributing factors, the promotion of physical activity, leaving specific advanced neurologic pharmacotherapies for selected cases^92^ (*Figure* *2*).

1. Given the intricate nature of medication schedules and the prevalence of medication‐related issues among individuals with HF and CI, physicians must meticulously assess and reconcile medication plans. Implementing strategies to enhance outcomes in this context may involve deprescribing, a supervised process of medication discontinuation. Candidate medications for deprescribing might include those known to exacerbate HF and/or listed in criteria such as the Beers criteria,^93^ which identifies medications with potential risks outweighing benefits in older adults, particularly concerning cognition. Additionally, treating co‐morbidities can further improve outcomes in this patient population (i.e., pressure control in hypertensive patients and rhythm control in AF patients.^94^
2. Despite the pleiotropic effect of exercise training in HF, including enhancements in exercise capacity, cardiac function, peripheral effects and quality of life, limited research has explored the impact of physical exercise on individuals with HF and MCI. Tanne et al. demonstrated cognitive improvements in 20 severe HF patients after an 18‐week supervised exercise programme.^95^ Redwine et al. reported similar enhancements in cognitive performance in 69 HF participants with tai chi and resistance band exercises over 16 weeks.^96^ However, Kitzman et al. did not observe cognitive improvements in 349 HF patients following a 12‐week rehabilitation programme (multi domain physical rehabilitation programme emphasizing strength, balance, mobility, and endurance).^97^ Conversely, Gary et al. found improved verbal memory in 69 HF participants with combined exercise and cognitive training over 3 months.^98^ The aforementioned studies have poorly defined interventions, and short follow‐up durations, thereby posing a high risk of bias.
3. New pharmacotherapeutic agents have emerged for addressing distinct categories of cognitive dysfunction. Cholinesterase inhibitors (such as donepezil, rivastigmine and galantamine) are presently advocated for managing MCI and dementia associated with AD. However, the utility of cholinesterase inhibitors in cognitive impairment unrelated to AD (for example in vascular CI) is not supported by evidence; this raises concerns regarding heightened risk of adverse effects (e.g., cardiac and gastrointestinal), alongside varying levels of evidence quality. Consequently, consensus guidelines do not advocate for the use of cholinesterase inhibitors in treating cognitive impairment not associated with AD due to the off‐label nature and lack of evidence‐based support for such usage.^99^ Recently, disease‐altering monoclonal antibodies have gained approval for treating individuals with MCI and dementia arising from AD. Phase 3 trials have demonstrated the efficacy of monoclonal antibodies (aducanumab‐avwa; lecanemab‐irmb) in diminishing amyloid‐beta plaques within the brain, resulting in moderate alleviation of CI.^100^, ^101^ However, both treatments are associated with an increased vulnerability to amyloid‐related imaging abnormalities, including cerebral microhemorrhages, cerebral macrohemorrhages, superficial siderosis, brain oedema, or sulcal effusion. Subjects with HF were excluded from these clinical trials, considering the widespread use of anticoagulation in HF patients. However, the benefit and targets of disease‐modifying treatment beyond classical AD is still ongoing and definitively need to be updated according to new findings and prospective results.

## Conclusions

In conclusion, the intricate relationship between HF and CI underscores the urgent need for a comprehensive approach to patient care. Both conditions are subject to similar disease processes and pathophysiological mechanisms, emphasizing their close interplay. The profound impact of CI on HF self‐care and independence further emphasizes the importance of addressing cognitive health in HF management, with significant implications for patient prognosis and quality of life. This accentuates the critical need for treatment strategies capable of addressing the cognitive dysfunction associated with HF, leading to improved patient brain health and well‐being.

## Conflict of interest

Mauro Massussi, Maria Giulia Bellicini, and Riccardo Proietti declare that they have no conflicts of interest relevant to the content of this work to disclose. Marianna Adamo received speaker fees from Abbott Vascular and Medtronic, outside of the submitted work. M. Metra received consulting honoraria for participation in steering committees or advisory boards or for speeches from Abbott Vascular, Amgen, AstraZeneca, Bayer, Edwards, Fresenius, Novartis, and Servier, outside of the submitted work. Alessandro Padovani has served on the scientific advisory board of GE Healthcare, Eli Lilly, and Actelion Pharmaceuticals; and has received speaker honoraria from Nutricia, IAM Pharmaceuticals, Lansgstone Technology, GE Healthcare, Eli Lilly, UCB Pharma, and Chiesi Pharmaceuticals, outside of the submitted work. Andrea Pilotto has served on the scientific advisory board of Z‐cube (technology division of Zambon Pharma) and has received speaker honoraria from Biomarin and Zambon Pharmaceuticals, outside of the submitted work.
